# Theranostic Applications of an Ultra-Sensitive *T*_1_ and *T*_2_ Magnetic Resonance Contrast Agent Based on Cobalt Ferrite Spinel Nanoparticles

**DOI:** 10.3390/cancers14164026

**Published:** 2022-08-20

**Authors:** Georgy Mikhaylov, Urska Mikac, Miha Butinar, Vito Turk, Boris Turk, Sergey Psakhie, Olga Vasiljeva

**Affiliations:** 1 Department of Biochemistry and Molecular and Structural Biology, Jozef Stefan Institute, SI-1000 Ljubljana, Slovenia; 2 Department of Condensed Matter Physics, Jozef Stefan Institute, SI-1000 Ljubljana, Slovenia; 3 Institute of Strength Physics and Materials Science, 634021 Tomsk, Russia; 4 CytomX Therapeutics, Inc., South San Francisco, CA 94080, USA

**Keywords:** cancer treatment, magnetic nanoparticles, magnetic resonance imaging, targeted drug delivery

## Abstract

**Simple Summary:**

Magnetic nanoparticles (MNPs) represent an important class of nanomaterials that has been actively employed in multiple technological applications. The MNPs and their based composites have been intensively developed for magnetic resonance imaging, targeted drug delivery, magnetic hyperthermia, and other applications. Magnetic Resonance Imaging (MRI) has a prominent position among clinical imaging modalities as it allows for high spatial resolution and tissue specificity without harmful ionizing radiation. The aim of the study was the demonstration of the potential use of magnetic nanoparticles based on cobalt ferrite spinel as advanced MRI contrast agents that are capable of both *T*_1_-weighted positive and *T*_2_-weighted negative contrast enhancements in vitro and in vivo. Furthermore, in the present study, we combined novel physical, chemical, and biomedical approaches to develop a multifunctional MRI-detectable drug delivery system that was an efficient *T*_1_- and *T*_2_-weighted MRI contrast agent and a nanocarrier for targeted drug delivery in vivo.

**Abstract:**

Nano-dimensional materials have become a focus of multiple clinical applications due to their unique physicochemical properties. Magnetic nanoparticles represent an important class of nanomaterials that are widely studied for use as magnetic resonance (MR) contrast and drug delivery agents, especially as they can be detected and manipulated remotely. Using magnetic cobalt ferrite spinel (MCFS) nanoparticles, this study was aimed at developing a multifunctional drug delivery platform with MRI capability for use in cancer treatment. We found that MCFS nanoparticles demonstrated outstanding properties for contrast MRI (*r*_1_ = 22.1 s^–1^mM^–1^ and *r*_2_ = 499 s^–1^mM^–1^) that enabled high-resolution *T*_1_- and *T*_2_-weighted MRI-based signal detection. Furthermore, MCFS nanoparticles were used for the development of a multifunctional targeted drug delivery platform for cancer treatment that is concurrently empowered with the MR contrast properties. Their therapeutic effect in systemic chemotherapy and unique MRI double-contrast properties were confirmed in vivo using a breast cancer mouse tumor model. Our study thus provides an empirical basis for the development of a novel multimodal composite drug delivery system for anticancer therapy combined with noninvasive MRI capability.

## 1. Introduction

Magnetic nanoparticles (MNPs) represent an important class of nanomaterials that has been actively employed in multiple technological applications over the past two decades [1,2,3,4,5,6]. Moreover, innovations in materials science and nanotechnology have advanced the evolution of MNPs from simple substances to metal oxides and alloys with superparamagnetic properties distinct from those in their bulk species counterparts [7,8]. The MNPs have been intensively developed for targeted drug delivery, magnetic resonance imaging (MRI), magnetic hyperthermia and thermoablation, bioseparation, and biosensing, among other applications [9,10]. In bio-applications, MNPs offer several unique advantages over traditional materials: they are relatively inexpensive to produce, physically and chemically stable, biocompatible, and environmentally safe [11].

Magnetic resonance imaging has a prominent position among clinical imaging modalities as it allows for high spatial resolution and tissue specificity based on varying relaxation times and proton densities between tissues without harmful ionizing radiation [12,13,14,15,16]. Thus, it provides safe and excellent soft tissue contrast for high spatial resolution imaging of structures deep within the body [16]. For more specific types of imaging, i.e., to enhance the contrast between normal and diseased tissues, MRI contrast agents are often used [17]. Iron oxide nanoparticles, such as magnetite (Fe_3_O_4_), maghemite (y-Fe_2_O_3_), and mixed ferrites (MFe_2_O_4_ where M = Co, Mn, Ni or Zn), are particularly promising MRI contrast agents due to their high saturation magnetization [18].

Nanostructures, such as Fe_3_O_4_@SiO_2_@HPG-FA and polyethylene glycol (PEG)-Arg@IONPs, have been successfully developed for MRI application in ex vivo and in vivo imaging studies [19,20]. Efremova et al. [21] developed magnetite-gold nanoparticles for in vivo diagnostics of a breast cancer model. They observed that nanoparticles accumulated inside the tumor 24 h after intravenous injection and enabled good MR contrast. Furthermore, Chen et al. and Islam et al. developed magnetic nanoprobes that enabled MRI and magnetic induction hyperthermia of a tumor using iron oxide (Fe_3_O_4_) and polysaccharide-chitosan-coated manganese ferrite (MnFe_2_O_4_), respectively [4,22].

The MRI contrast agents are divided into two groups: *T*_1_-weighted (positive) contrast agents, which increase signal intensity by shortening the longitudinal relaxation time of protons in tissues where they localize; and *T*_2_-weighted (negative) contrast agents, which yield lower MRI signals by shortening the transverse relaxation time of protons [23], thus providing a negative contrast [23,24]. The use of *T*_1_ contrast agents is suitable for in vivo applications due to a short repetition time (TR), which enables relatively short imaging times. Besides, *T*_1_ contrast agents do not produce any distortion in MR images. However, the performance of paramagnetic complexes based on gadolinium (Gd) is limited by rapid clearance from the blood into extravascular compartments, whereas *T*_2_ contrast agents are nanosized and remain intravascular for a prolonged period of time. It should be noted that conventional *T*_1_ MRI contrast agents based on metal oxides have a low relaxivity coefficient *r*_1_ that decreases their sensitivity on *T*_1_-weighted MR images [24,25,26,27,28]. On the other hand, *T*_2_ superparamagnetic contrast agents are associated with MR image distortion, and dark regions on *T*_2_-weighted images can easily be confused with other causes, such as calcification or metal deposits. A possible solution could be to develop MR contrast agents for deep tissue imaging that enable simultaneous detection of a probe on *T*_1_- and *T*_2_-weighted MR images [29,30,31,32], for which MNPs are considered promising candidates that enable high-resolution imaging and have suitable paramagnetic and physicochemical properties [4,23,25,33,34,35,36,37,38,39,40,41].

In this study, we present a contrast agent based on magnetic cobalt ferrite spinel (MCFS) nanoparticles with unique double-contrast properties enabling effective *T*_1_-weighted and *T*_2_-weighted MR imaging. We found that MCFS nanoparticles with enhanced *T*_1_ and *T*_2_ contrast properties could be effectively used for in vivo MRI applications and that this nano-dimensional material was more effective than other commonly used contrast agents. In addition, the MCFS nanoparticles were used for the development of a targeted drug delivery system, thus, empowered with a simultaneous *T*_1_- and *T*_2_-weighted MRI properties.

## 2. Materials and Methods

### 2.1. Preparation of MCFS

The MCFS nanoparticles were mechanochemically synthesized using saline crystal hydrates as previously described [8]. Sodium chloride, as an inert component, was added at a ratio of 1:2 to prevent heating during mechanical activation. The mixture was sealed by steel balls in a planetary mill, washed with distilled water, and dried at room temperature. Dry MCFS nanoparticles were suspended in a stabilizing buffer (20 mM sodium citrate buffer [pH 7.4] containing 108 mM NaCl and 10 mM HEPES). The resulting nanoparticle agglomerates were disrupted with an ultrasonic disintegrator (Branson Digital Sonifier SFX 550, Branson, Brookfield, CT, USA), followed by separation of the remaining undisrupted agglomerates by centrifugation at 500 g for 3 min (Eppendorf Centrifuge 5417C, Eppendorf, Hamburg, Germany). The nanoparticle concentration in the stabilizing buffer was measured using flame atomic absorption spectrometry on a Varian SpectrAA 110 (Varian, Mulgrave, Australia). The average size of non-aggregating nanoparticle clusters was characterized by dynamic light scattering (DLS) using a PDDLS/BatchPlus System (Precision Detectors, Bellingham, MA, USA). A FE-SEM SUPRA 35 VP (Carl Zeiss, Oberkochen, Germany) equipped with an Inca 400 energy dispersive spectroscope (Oxford Instruments, Abingdon, Oxfordshire, UK) was used for field emission gun scanning electron microscopy (FEG-SEM).

### 2.2. Preparation of MCFS Liposomes

The MCFS-loaded liposomes (MCFS-L) were prepared from 95% L-a-phosphatidylcholine (Avanti Polar Lipids, Birmingham, AL, USA) and 5% 1,2-distearoyl-sn-glycero-3-phosphoethanolamine-N-[methoxy(polyethylene glycol)-2000] (Avanti Polar Lipids, Birmingham, AL, USA) with a total lipid concentration of 2.75 mM. Organic solvent was evaporated in an Eppendorf Concentrator 5301 (Eppendorf, Hamburg, Germany) to generate dry lipid films, which were subsequently hydrated with MCFS nanoparticles in 20 mM citrate buffer (pH 7.4) containing 108 mM NaCl and 10 mM HEPES to generate multilamellar vesicles containing nanoparticles. The multilamellar vesicles were extruded by a mini-extruder with a polycarbonate membrane (pore size = 100 nm, Avanti Polar Lipids, Birmingham, AL, USA) to generate nanosized unilamellar bilayer liposomes. The size of the MCFS-L was characterized by DLS. Doxorubicin hydrochloride (Sigma-Aldrich, Burlington, MA, USA) was dissolved in MCFS nanoparticles containing stabilizing buffer and then encapsulated into liposomes. Nonencapsulated fraction of doxorubicyn was removed by gel filtration on sephadex column Sephadex^©^ G-75 (Sigma-Aldrich, Burlington, MA, USA) and final concentration of the encapsulated doxorubicin was detected by spectrophotometry. The efficacy of doxorubicin hydrochloride encapsulation was determined as 32%. The morphology of such liposome formulation and lipids composition was confirmed in the previous study by transmission electron microscopy (TEM) [42].

### 2.3. Cell Biocompatibility of MCFS Nanoparticles

Primary MMTV-PyMT cells were isolated and cultured as previously described [43]. The cells were maintained in DMEM supplemented with 10% fetal bovine serum (Sigma-Aldrich, Burlington, MA, USA), 2 mM L-glutamine (Invitrogen, Waltham, MA, USA), 100 units of penicillin, and 100 µg/mL streptomycin (Invitrogen, Waltham, MA, USA). Cultured cells were maintained at 37 °C in a humidified 5% CO2 atmosphere. Cells were incubated for 24 h with 55 mM MCFS nanoparticle solution (treatment group), phosphate buffer (pH 7.4, negative control), or 1 µM of staurosporine (STS, positive control). Phosphatidylserine exposure and membrane integrity were measured by labeling cells with annexin V-PE in the presence of propidium iodide according to the manufacturer’s instructions. Cells were then subjected to FACS analysis using a FACScalibur flow cytometer (Becton Dickinson, Franklin Lakes, NJ, USA) and CellQuest software (BD Bioscience, San Jose, CA, USA).

### 2.4. In Vitro and In Vivo MR Imaging

All MR experiments were performed using a TecMag Apollo MRI spectrometer with a superconducting 2.35 T (^1^H NMR frequency ν_H_ = 100 MHz) horizontal bore magnet (Oxford Instruments, Abingdon, Oxfordshire, UK) using a 25 mm saddle-shaped Bruker RF coil. Spin-lattice and spin-spin relaxation times (*T*_1_ and *T*_2_) were measured for different concentrations of MCFS nanoparticles in 1% agarose at room temperature using inversion recovery and spin-echo techniques, respectively. The longitudinal (*r*_1_) and transverse (*r*_2_) relaxivities were calculated from *r*_i_ = (1/*T*_i_–1/*T*_i0_)/c, where c is the concentration of MCFS nanoparticles in mM, *T*_i_ is the relaxation time at concentration c, *T*_i0_ is the relaxation time of 1% agarose, and i = 1 for *T*_1_ and 2 for *T*_2_. Two-dimensional (2D) MR images were recorded with a standard multislice spin-echo pulse sequence with an echo time (*TE*) of 8.5 and 60 ms and a *TR* of 400 and 2000 ms for *T*_1_- and *T*_2_-weighted MR images, respectively. The field of view was 40 mm with an in-plane resolution of 156 µm and a slice thickness of 1 mm.

For in vivo detection, an external magnet of 0.33 T was glued with cyanoacrylate to the right inguinal mammary gland of a 12-week-old mouse, and 200 µL of liposomes containing MCFS nanoparticles (0.15 mM) were administered intraperitoneally. The magnet was removed with acetone 1 h after the injection. The *T*_1_- and *T*_2_-weighted MR images were taken pre- and 24 h post-injection. During imaging, mice were anesthetized by subcutaneous injection of a ketamine-xylazine-acepromazine cocktail (50/10/1.0 mg/kg).

### 2.5. Acute Toxicity Study

Mice were sacrificed 14 d after injection of 500 mg/kg (*n* = 8), 1000 mg/kg (*n* = 8) of MCFS nanoparticles, or stabilizing buffer (*n* = 8) and serum was separated from blood collected at death by centrifugation in a Li-heparin 0.6 mL flask (Fuji Photo Film Co., Ltd., Life Science Products Division, Akasaka, Minato, Tokyo, Japan). Biochemical parameters were analyzed with a Fujifilm DRI CHEM 3500i (Fuji Photo Film Co., Ltd.) using biochemical slides from Fuji Photo Film Co., Ltd., as follows: for blood creatinine (CRE P-III), urea nitrogen (BUN P-III), creatine kinase (MB isozyme, CKMB P), lactate dehydrogenase (LDH P-III), alanine transaminase (GOT/ALT P-III), aspartate transaminase (GOT/AST P-III), alkaline phosphatase (ALP P-III), and α-amylase (AMYL P-III). The kidneys, spleen, liver, and lung were collected and fixed in 10% neutral formalin. Organs were dehydrated, embedded in paraffin blocks. The 5 µm sections were stained with Hematoxilyne and Eosine (Sigma-Aldrich, Burlington, MA, USA).

### 2.6. Animal Models

FVB/N-TgN(MMTVPyVT)634Mul mice were used in the present study and our experimental protocols were approved by the Administration of the Republic of Slovenia for Food Safety, Veterinary Sector and Plant Protection. Procedures for animal care and use were based on the PHS Policy on Human Care and Use of Laboratory Animals and the Guide for the Care and Use of Laboratory Animals (NIH publication 86–23, 1996). To generate tumors for a treatment study, primary PyMT tumor cells were obtained from 14-week-old MMTV-PyMT transgenic mice as previously described [43], culture-expanded, suspended in 200 µL serum-free Dulbecco’s Modified Eagle’s Medium (DMEM, Invitrogen, Waltham, MA, USA), and 5 × 10^5^ cells were injected into the left inguinal mammary gland of a recipient mouse (FVB/N mouse strain).

### 2.7. Treatment Study

Our dosing regimen for the doxorubicin treatment was determined based on the previous reports to reduce the cardiotoxic effect [44,45]. The single-injection treatment, followed by magnetic targeting, was administered when tumors reached a volume of 120 mm^3^. Doxorubicin was dissolved in MCFS nanoparticles containing stabilizing buffer and encapsulated in the PEGylated liposomes. The MCFS liposomes loaded with doxorubicin (2 mg of doxorubicin per 1 mL of liposomes) were intraperitoneally administered at a dose of 12 mg/kg (Dox/MCFS-L, *n* = 8). As described in Section 2.3, 0.3 T magnets (5 mm diameter) were attached to the tumor before injection and removed 24 h later. The experimental groups were treated with stabilizing buffer (control, *n* = 8), MCFS-L with magnetic targeting (MCFS-L, *n* = 8), or doxorubicin (Dox, *n* = 8). The horizontal and vertical tumor diameters were measured every second day until the end of treatment with a digital caliper and tumor volume was calculated as V = (a × b^2^) ∗ π/6, where a and b are the longer and shorter diameters of the tumor, respectively. Mice were sacrificed on day 8 post treatment.

### 2.8. Statistical Analysis

Data are presented as the mean ± standard error of the mean (SEM). The differences in the treatment effect were compared using a Student’s *t*-test and statistical significance was set at *p* ≤ 0.05.

## 3. Results

### 3.1. Development and Characterization of MCFS Nanoparticles

**Figure 1:**
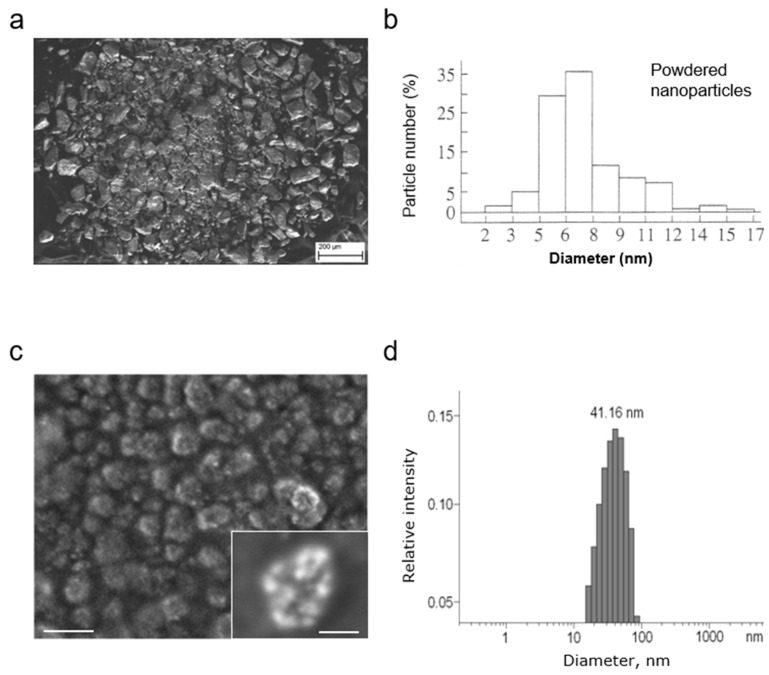
Characterization of the magnetic cobalt ferrite spinel (MCFS) nanoparticles. (**a**), Scanning electron micrographs of MCFS nanoparticles. (**b**), Size distribution of MCFS nanoparticles (average diameter = 7.99 nm). (**c**), Field emission gun scanning electron microscopy of the aqueous colloidal MCFS nanoparticles. (**d**), Distribution of nanoparticle cluster diameters and their average size (diameter = 41.16 nm) according to dynamic light scattering measurements.

The mechanochemically synthesized MCFS nanoparticles were 2–17 nm in diameter, >70% of which were less than 8 nm (Figure 1a,b), with a specific surface area of 150 m^2^/g. The generated nanoparticles acquired the characteristic features of superparamagnetic state or cluster spin-glass behavior. Moreover, the decrease of the structural element size to 2–17 nm greatly improved certain magnetic properties, such as the specific saturation magnetization (26 G·cm^3^/g) and strength of the anisotropy field (520 Oe). Finally, partial substitution of iron with cobalt in the magnetite matrix improved the magnetic moment of metal oxide.

The main limiting factor in using magnetic nanoparticles in vivo is their low colloidal stability. Therefore, to prevent their agglomeration, we employed an optimized two-step procedure for preparing a biocompatible aqueous colloidal system from powdered MCFS nanoparticles, which produces a narrower particle size distribution of nanoclusters [42] (Figure 1c). The concentration of MCFS nanoparticles was measured by flame atomic absorption spectrometry, and the unit average size of nanoparticles was determined by dynamic light scattering (DLS) (Figure 1d). The average size of stabilized non-aggregated clusters determined by DLS was 41.16 nm (Figure 1d). The resulting MCFS nanoparticles had a negative surface zeta potential 28.4 ± 2.2 mV at pH 7.4 and 37.2 °C.

### 3.2. MR Contrast Properties of MCFS Nanoparticles In Vitro

**Figure 2:**
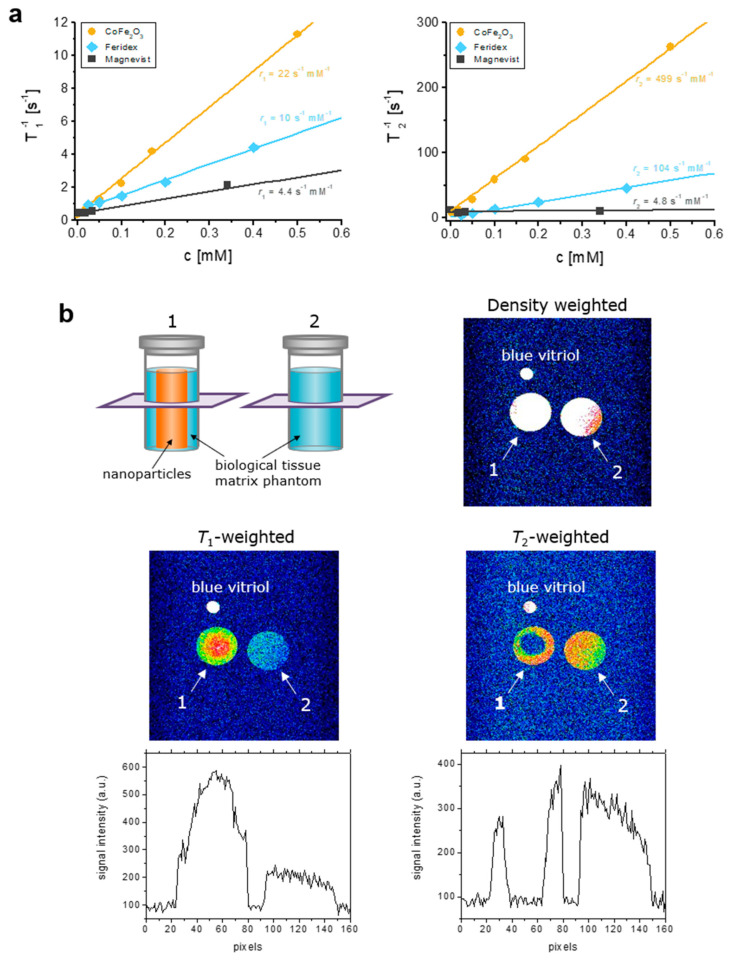
MR contrast properties of biocompatible MCFS nanoparticles with improved biocompatibility. (**a**), Spin-lattice 1/*T*_1_ and spin-spin 1/*T*_2_ relaxation rates of MCFS nanoparticles at different concentrations, compared to commercially available MR contrast agents in 1% agarose. Symbols are measured values, and lines are fit to the equation 1/*T*_i_ = *r*_i_·*c* + 1/*T*_i0_, where *r_i_* is the relaxivity, *c* is the concentration, *T_i_*_0_ is the relaxation rate of 1% agarose, and *i* is 1 for *T*_1_ and 2 for *T*_2_. Relaxivity rates *r_1_* and *r_2_* were obtained by comparison of measured and theoretical values. (**b**), Schematic representation of agarose phantom models with density (*TE* = 8.5 ms, *TR* = 2000 ms), *T*_1_*-* (*TE* = 8.5 ms, *TR* = 400 ms), and *T*_2_-weighted MR images (*TE* = 60 ms, *TR* = 2000 ms) of two phantom-probes containing 1% agarose (2) and 0.15 mM MCFS nanoparticles placed in the center of the 1% agarose gel probe (1) along with signal intensity profiles.

To evaluate MR contrast properties, the different concentrations of MCFS nanoparticles were scanned at *T*_1_ and *T*_2_ relaxation times. The respective relaxivity coefficients were *r*_1_ = 22.1 s^–1^mM^–1^ and *r*_2_ = 499 s^–1^mM^–1^. Compared to our MCFS nanoparticles, commercially available *T*_1_ and *T*_2_ MR contrast agents Magnevist (Bayer, Leverkusen, Germany) and Ferridex (Bayer, Leverkusen, Germany) demonstrated the lower relaxivity coefficients and poorer *T*_1_ and *T*_2_ MRI contrasts (Figure 2a).

To evaluate the efficacy of MCFS nanoparticles as *T*_1_ and *T*_2_ MRI contrast agents for in vivo applications, we employed a tissue matrix phantom model based on agarose gel that has similar MR properties to those of fat and tumor tissue [46]. The respective phantom-probes were developed by placing 1% agarose in a glass flask and injecting the nanoparticle agarose solution at its center (Figure 2b). The respective control and testing phantom-probes were then subjected to *T*_1_- and *T*_2_-weighted MR scanning. Notably, on the *T*_1_-weighted MR image, a bright signal enhancement was observed in the middle of test sample #1 (Figure 2b), whereas a negative contrast was visualized at the same site on the *T*_2_-weighted image (Figure 2b). These results demonstrate the effective dual *T*_1_ and *T*_2_ contrast MRI properties of MCFS nanoparticles that could enhance the diagnostic capabilities and detection limits of traditional MR techniques.

### 3.3. Safety and Toxicity of MCFS Nanoparticles

**Figure 3:**
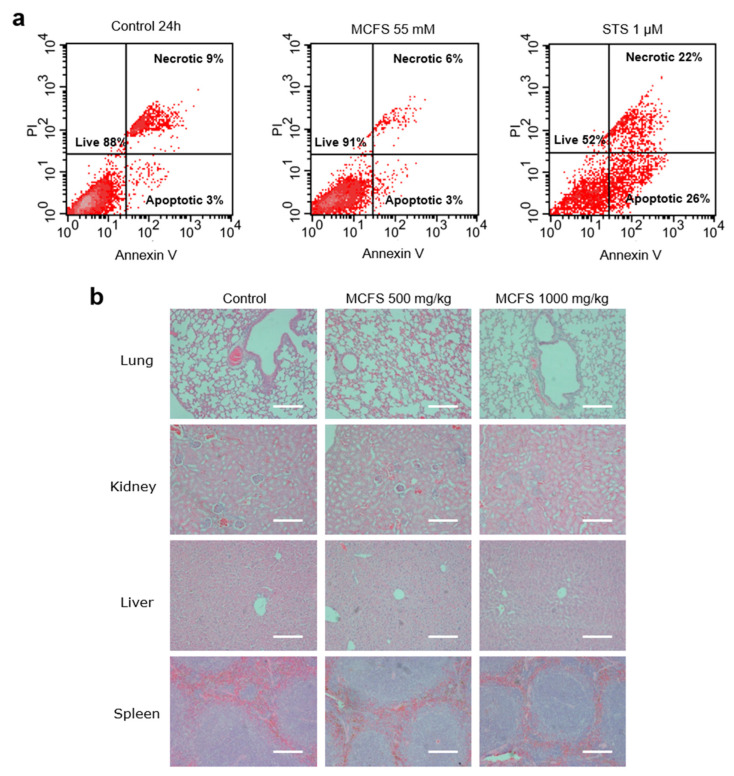
Assessment of the toxicity of MCFS nanoparticles. (**a**), Flow cytometry of MMTV-PyMT mouse breast cancer cells either untreated (control), treated with 55 mM of MCFS nanoparticles, or 1 µm of STS in the presence of annexin V-PE and stained with propidium iodide. (**b**), Hematoxylin and eosin staining of tissues from mice treated with different concentrations of MCFS. Lung, kidney, liver, and spleen collected from mice 14 d after injection of 500 mg/kg MCFS or 1000 mg/kg MCFS nanoparticles in stabilizing buffer. Samples were dehydrated, embedded in paraffin, sliced to 5 µm sections, and stained by hematoxylin and eosin. Scale bar = 100 μm.

The safety of MCFS nanoparticles was tested both ex vivo and in vivo. First, we incubated primary tumor cells isolated from a genetically engineered mouse model of human breast cancer (MMTV-PyMT) for 24 h with 55 mM of MCFS nanoparticles. We found no difference in the rates of cell death between the control and MCFS cell cultures (Figure 3a). Next, to investigate possible adverse effects of MSCF nanoparticles, an acute toxicity experiment was conducted using FVB mice treated for 14 d with 500 mg kg^–1^ or 1000 mg kg^–1^ MCFS nanoparticles. No adverse effects were detected for either of the treatment concentrations and no changes in blood biochemistry profiles or tissue histopathology were observed between the MCFS treated and control group (Figure 3b, Table 1).

### 3.4. MCFS Nanoparticles as an MRI-Visible Drug Delivery System In Vivo

**Figure 4:**
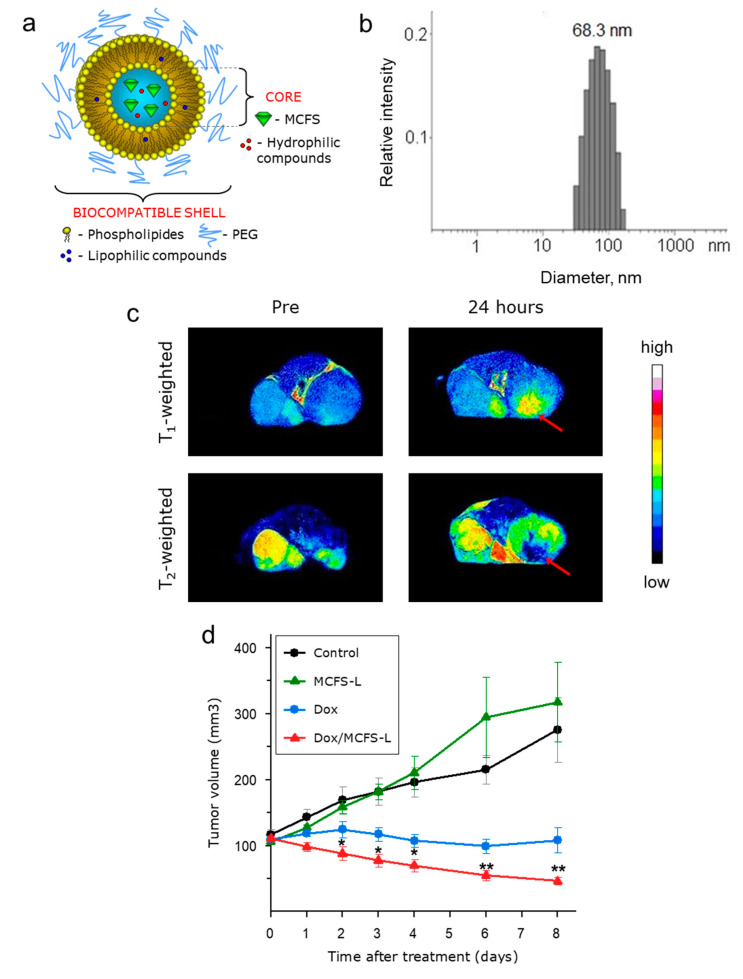
Multifunctional use of MCFS nanoparticles encapsulated in PEGylated liposomes. (**a**), Schematic representation of the biocompatible shell-forming liposome containing MCFS nanoparticles. (**b**), Dynamic light scattering measurement of MCFS-L showing the distribution of liposome diameters and average size (diameter = 68.3 nm). (**c**), In vivo *T*_1_- (*TE* = 8.5 ms, *TR* = 400 ms, slice thickness = 1 mm) and *T*_2-_weighted MR images (*TE* = 60 ms, *TR* = 2000 ms, slice thickness = 1 mm) of an MMTV-PyMT transgenic mouse before and 24 h after intraperitoneal administration of 200 µL MCFS-L solution (0.15 mM) followed by 1 h of magnetic field application. The clear brightening in the *T*_1_-weighted opposite the homogeneous darkening on the *T*_2_-weighted images in the areas of the tumor exposed to the 0.3 T magnet (red arrow) indicate preferential accumulation of MCFS-L. (**d**), Dynamics of tumor growth after single-injection treatment of mice by Dox-MCFS-L (*n* = 8), systemic doxorubicin (*n* = 8), nanoparticles in stabilizing buffer (*n* = 8), and MCFS-L (*n* = 8). Treatment was performed after the tumor volume reached 120 mm^3^. * *p*, 0.05 and ** *p*, 0.01, compared with the other groups.

Lipid vesicles, such as liposomes, are highly compatible with biological membranes in both composition and structure, thus supporting their extensive applications to a variety of drug delivery systems [42,47,48,49,50,51,52]. Several liposome-based systems loaded with magnetic nanoparticles, also called magneto-liposomes, have recently been developed for targeted delivery of drugs to a tumor [42,47,53,54,55]. To enable the targeted delivery of simultaneously diagnostic and therapeutic agents to a cancer site with their simultaneous MRI detection, we encapsulated the MCFS nanoparticles into sterically stabilized PEG-coated nanosized stealth liposomes. Thereby formed MCFS liposomes (MCFS-L) could be targeted by magnetic field and at the same time enable MRI monitoring of their distribution (Figure 4a). The liposome surface was PEGylated to reduce opsonization and clearance of the MCFS-L by the reticuloendothelial (mononuclear phagocyte) system (Figure S1) [42]. The MCFS-L was non-toxic to cells and appeared at an average diameter of 68.3 nm as measured by DLS (Figure 4b and Figure S2).

We assessed the efficiency of the MCFS-L in vivo in a genetically engineered mouse model of human breast cancer (MMTV-PyMT) with multifocal mammary adenocarcinomas [56]. First, magnetic targeting of the MCFS-L was evaluated by intraperitoneal administration into an MMTV-PyMT tumor-bearing mouse while a magnetic field was applied for 1 h to the left of the inguinal mammary tumor. Targeting of the MCFS-L was assessed by *T*_2_- and *T*_1_-weighted MRI scans (Figure 4c). As expected, the MCFS nanoparticles delivered by liposomes appeared as a dark area on *T*_2_- and enhanced signal on *T*_1_-weighted images 24 h post-injection (Figure 4c). These data confirm successful targeting of the MCFS-L system to the tumor region with concurrently enabled double-contrast MRI capability.

Next, we evaluated the targeted drug delivery capabilities of the MCFS-L using a standard cancer chemotherapy drug, doxorubicin. To overcome the difficulties in measuring tumors in the transgenic MMTV-PyMT mouse model with multifocal mammary tumors and to secure the functional immune system (as compared to a xenograft approach), an orthotopically transplanted mouse mammary tumor model was developed by inoculating 5 × 10^5^ primary MMTV-PyMT tumor cells into the mammary gland of a congenic immunocompetent recipient mouse (FVB/N mouse strain). The doxorubicin-loaded MCFS-L was administered intraperitoneally with a magnetic field focused on the tumor and its anti-tumor effect was compared to targeted MCFS-L non-loaded with doxorubicin, systemic administered doxorubicin, and no treatment control animals (Figure 4d). Notably, a single-dose treatment of doxorubicin targeted by MCFS-L resulted in a 41% reduction in tumor volume 8 d after administration, while a standard doxorubicin administration only achieved tumor stasis (Figure 4d). Collectively, these results demonstrate the potential of the MCFS-L for a variety of therapeutic and imaging applications.

## 4. Conclusions

Magnetic drug targeting, i.e., using magnetic nanoparticles loaded with therapeutic agents and an external magnetic field focused on the target tissue, has previously demonstrated promising results in animal tumor models. In our previous study, we successfully targeted the cathepsin inhibitor JPM-565 in a mouse breast cancer model with ferri-liposomes based on Fe_3_O_4_ nanoparticles through the externally applied magnetic field, which resulted in a significant reduction in tumor growth [42,57]. Similarly, magnetically targeted polymeric micelles loaded with iron nanoparticles and celastrol showed superior anti-tumor activity in vivo through inhibition of NF-κB activation, VEGF, and COX-2 [58]. In addition, magnetic nanoparticles have gained additional attention because of their unique MRI contrast properties and could thus be applied to noninvasive in vivo MRI. In a previous study, magnetic nanoparticles were functionalized with folate for diagnostic MRI application and early breast cancer detection [19]. In addition, protein coating of electrostatically stabilized nanoparticles with bovine serum albumin significantly improved dispersion stability in the presence of an increasing concentration of NaCl solution. Thus, albumin-coated MNPs are a particularly promising candidate for further research in MRI molecular imaging [59]. In the addition to the functional assays and in vivo efficacy studies, the release of encapsulated compound in tumor microenvironment was demonstrated in our previous study utilizing double transgenic mice (FVB.luc^tg/+^; PyMT^tg/+^) that develop breast tumors with simultaneous expression of luciferase throughout the body. After administration and targeting of ferri-liposomes loaded with the luciferase substrate (D-luciferin) to the tumor, a luminescent signal was detected exclusively in the tumor exposed to the magnet, indicating both the effective tumor targeting and the release of the cargo from the targeted liposomes in vivo [42].

In the present study, we combined novel physical, chemical, and biomedical approaches to develop a multifunctional MRI-detectable drug delivery system that was an efficient *T*_1_- and *T*_2_-weighted MRI contrast agent and nanocarrier for targeted drug delivery in vivo. Our results demonstrated the potential use of MCFS nanoparticles as advanced MRI contrast agents that are capable of both *T*_1_-weighted positive and *T*_2_-weighted negative contrast enhancements in vitro and in vivo. The developed MCFS nanoparticles demonstrated higher *r*_1_ and *r*_2_ relaxivities and improved sensitivity in *T*_1_- and *T*_2_-weighted MR images compared to other nanoparticles or paramagnetic complexes based on Gd, such magnesium oxide or Resovist^®^ [25,26]. Moreover, compared to conventional *T*_1_ and *T*_2_ contrast agents, such as Magnevist^®^ and Ferridex, MCFS demonstrated a remarkable diversity in relaxation time, thus demonstrating superior performance as *T*_1_ and *T*_2_ MRI contrast agents.

Taken together, MCFS nanoparticles could serve as an ultra-sensitive *T*_1_ and *T*_2_ double-contrast agent that enables a clearer distinction between cancerous and healthy tissues. Furthermore, MCFS nanoparticle-based liposomes represent an effective multimodal drug delivery platform that can encapsulate a wide range of therapeutic agents and, combined with magnetic targeting, offer multiple diagnostic and therapeutic opportunities for tumor targeting with simultaneous MRI detection functionality. Such properties could be particularly valuable for reduction of side effects of cytotoxic drugs and increase the precision of cancer treatment.

We have shown here a novel nanoplatform for the therapy and imaging of cancer which is based on magnetic cobalt ferrite spinel (MCFS) nanoparticles with unique dual-contrast properties enabling effective *T*_1_- and *T*_2_-weighted MR imaging. We found that MCFS nanoparticles demonstrated efficient anticancer effects as a targeted drug delivery system through the externally applied magnetic field, and outstanding properties for high-resolution *T*_1_- and *T*_2_-weighted MRI-based signal detection. A simultaneous therapeutic effect in chemotherapy drug delivery and unique MRI double-contrast properties were confirmed in vivo using a breast cancer mouse tumor model. We believe that our study makes a significant contribution to the literature as it provides an empirical basis for the development of a novel multimodal composite drug delivery system for anticancer therapy combined with non-invasive MRI capability.

## 5. Patents

Psakhye S., Itin V., Magaeva A., Nayden E., Vasiljeva O., Mikhaylov G., Mikac M., Turk B. Contrast agent for t1 and/or t2 magnetic resonant scanning and method for preparing it. RU2471502C1. 2013. RU (1).

## Figures and Tables

**Table 1:** Renal, cardiac, liver, and pancreatic functions in mice after systemic infusion of MCFS nanoparticles.

Treatment Groups, Dose	Renal Function		Cardiac Function		Liver Function		Pancreatic Function	
	Creatinine µmol/L	Urea Nitrogen mmol/L	Creatine Kinase (MB) U/L	Lactate Dehydrogenase U/L	Alkaline Phosphatase U/L	Alanine Transaminase U/L	Aspartate Transaminase U/L	α-Amylase U/L
Control, Males	19 ± 3.8	10 ± 0.3	229.8 ± 61	1129 ± 295	99.7 ± 16.4	43.7 ± 4.8	96 ± 11.7	2281 ± 268
Control, Females	46 ± 7.5	13.3 ± 0.3	242.2 ± 133	960.2 ± 213	113.7 ± 8.2	59.5 ± 4	87.5 ± 23.1	2620 ± 373
500 mg/kg, Males	16 ± 2.1	10 ± 0.8	185.2 ± 111	711.7 ± 62	97.2 ± 10.8	43.2 ± 3.94	66.5 ± 7.9	2976 ± 248
500 mg/kg, Females	37.7 ± 1.1	12 ± 0.6	72.7 ± 6.1	586 ± 21.8	104 ± 7.5	68.7 ± 7.3	58.2 ± 3.7	3157 ± 11
1000 mg/kg, Males	19.5 ± 1.5	8.8 ± 1	239.7 ± 93	1191 ± 269	82 ± 10	60.5 ± 22.8	84.5 ± 26.6	2685 ± 117
1000 mg/kg, Females	51.7 ± 8.3	12.1 ± 0.2	95 ± 19.7	706.7 ± 63	78.2 ± 6.5	52 ± 8.3	63.5 ± 5.1	2986 ± 236

## Data Availability

The data presented in this study are available on request from the corresponding author.

## Supplementary Materials

The following supporting information can be downloaded at https://www.mdpi.com/article/10.3390/cancers14164026/s1. Figure S1: Effectiveness of liposome pegylation for masking them from the macrophages uptake. Figure S2: Assessment of MCFS-Lip toxicity on cells.
