# Supplementary material for: Theranostic Applications of an Ultra-Sensitive T1 and T2 Magnetic Resonance Contrast Agent Based on Cobalt Ferrite Spinel Nanoparticles
Source: Cancers (Basel). 2022 Aug 20;14(16):4026. doi: 10.3390/cancers14164026 (PMC9406498; doi:10.3390/cancers14164026)
Supplement: Supplementary file 1 [file cancers-14-04026-s001.zip › cancers-1845369-supplementary.pdf]

SUPPLEMENTARY INFORMATION

THERANOSTIC APPLICATIONS OF AN ULTRA-SENSITIVE T_1 AND T_2 MAGNETIC RESONANCE CONTRAST AGENT BASED ON COBALT FERRITE SPINEL NANOPARTICLES

Georgy Mikhaylov[1], Urska Mikac[2], Miha Butinar[1], Vito Turk[1], Boris Turk[1], Sergey Psakhie[3, ‡], Olga Vasiljeva[1, §, *]

[1]Department of Biochemistry and Molecular and Structural Biology, Jozef Stefan Institute, Ljubljana, SI-1000, Slovenia

[2]Department of Condensed Matter Physics, Jozef Stefan Institute, Ljubljana, SI-1000, Slovenia

[3]Institute of Strength Physics and Materials Science, Tomsk 634021, Russia,

[‡]Deceased

[§]Present address: OV: CytomX Therapeutics, Inc., South San Francisco, CA 94080, USA

[*]E-mail: olga.vasiljeva@ijs.si

SI Materials and Methods

Effectiveness of liposome pegylation for masking them from the macrophages uptake.

THP-1 cells were grown at 37 °C in a humidified air atmosphere with 5% CO_2. The cells were cultured in the RPMI 1640 medium, supplemented with 10% fetal calf serum, 2 mM l-glutamine, 100 U/ml penicillin, 100 µg/ml streptomycin, and 10 mM HEPES buffer. For differentiation of THP-1 cells to macrophages-like phenotype 30 µl of 10 µM, phorbol 12-myristate 13-acetate (PMA, Invitrogene, Waltham, Massachusetts, USA) was added. 24 hours after cells were incubated with 100 µl of Alexa Fluor 546™ functionalized pegylated and non-pegylated liposomes in Dulbecco's Modified Eagle Medium (DMEM, Waltham, Massachusetts, USA) on 96-well optical bottom plate (Nunc, Roskilde, Denmark). After 15 minutes of incubation, cells were washed 3 times by phosphate buffer pH 7.4 and examined by 96-well TECAN plate reader at 561 nm excitation and 572 nm emission (Tecan, Männedorf, canton of Zürich, Switzerland).

Cell culture and toxicity ex vivo.

Primary MMTV-PyMT cells were isolated and cultured as described[1]. The cells were maintained in DMEM supplemented with 10% fetal bovine serum (Sigma), 2 mM L-glutamine (Invitrogene, Waltham, Massachusetts, USA), 100 units of penicillin, and 100 µg/ml streptomycin (Invitrogen) at 37°C in a humidified 5% CO_2 atmosphere. Cells were incubated for 24 hours with 0.15 mM MCFS-Lip solution and with PBS buffer for negative control. As a positive control, 1 µM of staurosporine (STS) was added. Phosphatidylserine exposure and the loss of membrane integrity were measured by labeling cells with Annexin V-PE in the presence of propidium iodide according to the manufacturer's instructions. Cells were then

subjected to FACS analysis using FACScalibur flow cytometer (Becton Dickinson, Franklin Lakes, NJ, USA) and CellQuest software.

References

1. Vasiljeva, O.; Papazoglou, A.; Krüger, A.; Brodoefel, H.; Korovin, M.; Deussing, J.; Augustin, N.; Nielsen, B.S.; Almholt, K.; Bogyo, M. Tumor cell-derived and macrophage-derived cathepsin B promotes progression and lung metastasis of mammary cancer. *Cancer research* **2006**, *66*, 5242-5250.

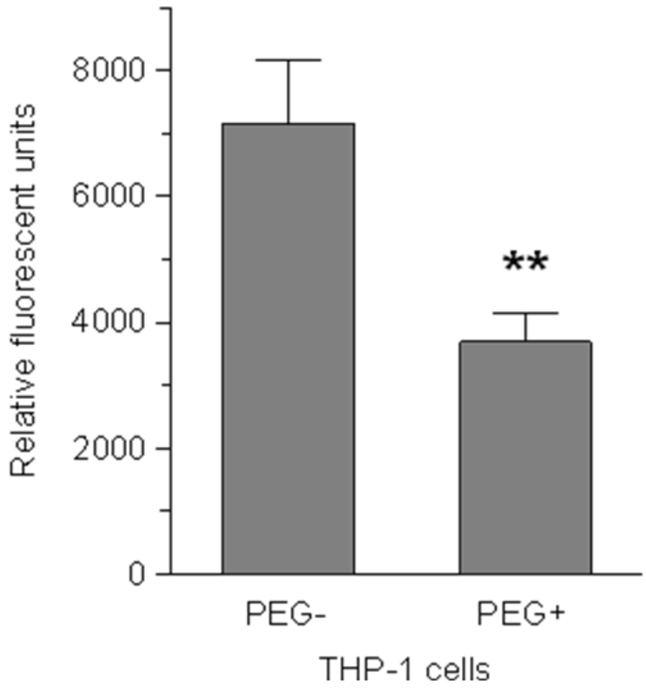

Figure S1.

Effectiveness of liposome pegylation for masking them from the macrophages uptake. Fluorescence intensity from the THP-1 cells differentiated to the macrophages-like phenotype and incubated with pegylated and non-pegylated liposomes loaded by Alexa Fluor 565™ (** $p<0.01$).

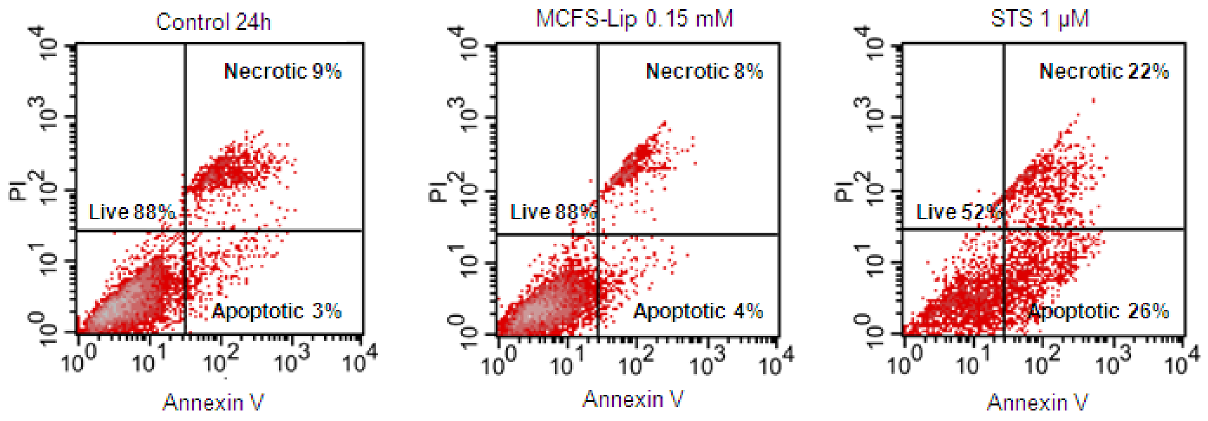

Figure S2. Assessment of MCFS-Lip toxicity on cells.

PyMT mouse breast cancer cells labeled by Annexin V-PE in presence of 5 µg/ml of propidium iodide. Cells were untreated (negative control), treated with 0.15 mM of MCFS-Lip, and treated 1 µm of STS (positive control). The intensity of fluorescence was measured by flow cytometry and data analyzed by Cell Quest software.
